# Impact of Fruit and Vegetable Enzyme Supplementation on Aerobic Performance and Lactate Response in Older Adults Following High-Intensity Interval Exercise Through Exergaming: Randomized Experimental Matched-Pair Study

**DOI:** 10.2196/52231

**Published:** 2024-06-25

**Authors:** Shu-Cheng Lin, Chien-Yen Wang, Tien-Hung Hou, Hong-Ching Chen, Chia-Chi Wang

**Affiliations:** 1 Department of Sport, Leisure and Health Management, Tainan University of Technology, Tainan, Taiwan; 2 Department of Athletics, National Taiwan University, Taipei, Taiwan; 3 General Education Center & Department of Regimen and Leisure Management, Tainan University of Technology, Tainan, Taiwan; 4 Physical Education Office, National Taipei University of Business, Taipei, Taiwan

**Keywords:** Ring Fit Adventure, training load, older adult training, training impulse, food supplement, older adults, exergames, exergame, Taiwan, female, fruits, vegetables, blood lactate, exercise, feasibility, aerobic, enzymes, enzyme, female older adults, fitness, food intake, diet, exergaming, enzyme supplements, older adults training, female older adult, older adult

## Abstract

**Background:**

Exercise offers substantial health benefits but can induce oxidative stress and inflammation, especially in high-intensity formats such as high-intensity interval exercise (HIIE). Exergaming has become an effective, enjoyable fitness tool for all ages, particularly older adults. Enzyme supplements may enhance exercise performance by improving lactate metabolism and reducing oxidative stress.

**Objective:**

This study investigates the efficacy of fruit and vegetable enzyme supplementation in modulating fatigue and enhancing aerobic capacity in older adults following HIIE through exergaming.

**Methods:**

The study recruited 16 older adult female participants and allocated them into 2 distinct groups (enzyme and placebo) based on their pretest lactate levels. This division used pairwise grouping to guarantee comparability between the groups, ensuring the integrity of the results. They engaged in HIIE using Nintendo Switch Ring Fit Adventure, performing 8 sets of 20 seconds of maximum effort exercise interspersed with 30 seconds of rest, totaling 370 seconds of exercise. Key metrics assessed included blood lactate levels, heart rate, rating of perceived exertion, and training impulse. Participants in the enzyme group were administered a fruit and vegetable enzyme supplement at a dosage of 30 mL twice daily over a period of 14 days.

**Results:**

The enzyme group showed significantly lower blood lactate levels compared to the placebo group, notably after the fourth (mean 4.29, SD 0.67 vs mean 6.34, SD 1.17 mmol/L; *P*=.001) and eighth (mean 5.84, SD 0.63 vs mean 8.20, SD 1.15 mmol/L; *P*<.001) exercise sessions. This trend continued at 5 minutes (mean 6.85, SD 0.82 vs mean 8.60, SD 1.13 mmol/L; *P*=.003) and 10 minutes (mean 5.91, SD 1.16 vs mean 8.21, SD 1.27 mmol/L; *P*=.002) after exercise. Although both groups exceeded 85% of their estimated maximum heart rate during the exercise, enzyme supplementation did not markedly affect the perceived intensity or effort.

**Conclusions:**

The study indicates that fruit and vegetable enzyme supplementation can significantly reduce blood lactate levels in older adults following HIIE through exergaming. This suggests a potential role for these enzymes in modulating lactate production or clearance during and after high-intensity exercise. These findings have implications for developing targeted interventions to enhance exercise tolerance and recovery in older adults.

## Introduction

Exercise represents a paradoxical element in health management, offering substantial benefits yet posing potential risks if not properly moderated [1,2]. High-intensity exercise, although efficacious in improving various health parameters, can lead to oxidative stress, muscle damage, and inflammation [3,4]. The oxidative stress primarily arises from increased reactive oxygen species production during intensive physical activities [5]. Moreover, exercise-induced fatigue serves as a protective mechanism against overexertion and consequent injuries [6,7]. In contemporary fitness regimes, high-intensity interval exercise (HIIE), particularly the Tabata training method, has gained prominence for its effectiveness in enhancing aerobic power, fat oxidation, and muscular endurance [8-10]. These attributes are especially crucial for the older adult population, a demographic that significantly benefits from regular physical activity [11-13].

Exergaming, an innovative blend of physical exercise and interactive gaming, has emerged as a transformative approach to fitness, especially in engaging diverse age groups in regular physical activity. Its efficacy in enhancing key fitness parameters such as aerobic capacity, agility, and coordination, coupled with its ability to make exercise more enjoyable, has been well documented [14-16]. This fusion of technology and exercise not only caters to the digital age but also opens avenues for personalized fitness experiences, which are adaptable to various demographic needs [17,18]. Although exergaming has been effective across a range of ages, its application in older adult populations presents unique opportunities and challenges. As the older adult population seeks safe, engaging, and effective exercise methods, exergaming could offer a solution that aligns with these requirements. However, integrating HIIE concepts into exergaming for older adults remains a relatively uncharted territory. HIIE, known for its efficiency in improving cardiovascular health and metabolic function, could significantly benefit older adults, particularly in terms of enhancing functional capacity and overall quality of life [12,19].

The potential of HIIE within exergaming for older adults hinges on the balance between intensity and safety. Although HIIE is beneficial, it is crucial to adapt its intensity to suit the physiological capabilities and limitations of older individuals. Research indicates that tailored HIIE programs can be both feasible and beneficial for older adults, leading to improvements in cardiovascular health, muscle strength, and metabolic function [20,21]. Integrating these concepts into exergaming could further enhance adherence and enjoyment, which are crucial factors in maintaining regular exercise habits in this demographic. Furthermore, the interactive and immersive nature of exergaming can address common barriers to exercise among older adults, such as the lack of motivation or fear of injury. By providing a safe, controlled environment for engaging in HIIE, exergaming can potentially transform the perception and experience of high-intensity workouts for older adults. This is particularly pertinent given the increasing need for innovative exercise interventions that cater to the aging global population [11].

Nutritional supplementation, especially with natural fruit and vegetable enzymes, presents a promising avenue in augmenting exercise performance through their antioxidant, anti-inflammatory, and metabolic benefits [21-28]. Such supplementation could potentially optimize lactate metabolism and enhance muscle function during exercise. Recent advancements in nutritional science have highlighted the substantial role of natural fruit and vegetable enzymes in enhancing exercise performance. These enzymes are increasingly recognized for their multifaceted health benefits, including their antioxidant, anti-inflammatory, and metabolism-enhancing properties [21,22]. Notably, their potential impact on exercise physiology, particularly in the context of high-intensity workouts, offers a new perspective on improving athletic performance and recovery.

One of the critical areas where these enzymes show promise is in the modulation of lactate metabolism. Lactate, often produced in higher quantities during intense physical activity, can lead to fatigue and decreased muscle efficiency. The traditional view of lactate as merely a byproduct of anaerobic metabolism has evolved, with current research acknowledging its role as a valuable energy source during prolonged exercise [23]. This shift in understanding opens up new avenues for using enzyme supplementation to optimize lactate use. Enzymes such as bromelain and papain, found in pineapples and papayas, respectively, have been studied for their potential in improving lactate metabolism. These enzymes are known to facilitate faster clearance of lactate from the bloodstream, thereby enhancing recovery and reducing fatigue [26,28]. Furthermore, the antioxidant properties of these enzymes play a crucial role in combating oxidative stress, which is often elevated during intense exercise regimens [24,25]. This reduction in oxidative stress is not only beneficial for immediate recovery but also contributes to long-term muscle health and function. Moreover, the anti-inflammatory actions of these natural enzymes can mitigate the inflammatory response often triggered by high-intensity exercise [27]. By reducing inflammation, these enzymes may enhance muscle recovery and function, thus allowing for more efficient and prolonged exercise performance. This aspect is particularly relevant in training regimens where recovery is as crucial as the exercise itself.

The primary aim of this feasibility study is to examine the effects of fruit and vegetable enzyme supplementation on aerobic capacity and blood lactate response in older adults engaged in HIIE through an exergaming framework. This study is dual faceted, focusing on (1) the physiological responses and feasibility of an exergaming HIIE regimen tailored for older adults and (2) the impact of enzyme supplementation on enhancing these exercise outcomes.

## Methods

### Sample Size

The sample size computation was based on the study by Flanagan and Jakeman [29]. Based on a statistical power analysis, a total sample size of 16 participants (8 per group) was needed to achieve a statistical power of 0.8 to detect a large effect size (ES) for supplement-time interaction at an α level of .05 [30].

### Participants and Experimental Design

After recruiting a total of 30 healthy older adult participants, the study proceeded with screenings and initial explanations. Subsequently, 12 individuals were excluded as they did not meet the inclusion criteria, and 2 declined to participate. Ultimately, 16 female older adult participants were enrolled in the study. These participants were then divided into 2 distinct groups (enzyme and placebo) based on their pretest lactate levels. Pairwise grouping was used to ensure comparability between the groups, thereby preserving the integrity of the results. All participants reported a regular exercise habit (3 times per week within the past year). They also completed the Physical Activity Readiness Questionnaire and confirmed no history of upper-limb skeletal muscle injury or major injury. Participants were instructed to avoid strenuous activities and the intake of caffeine or muscle-enhancing supplements for 24 hours prior to the experiment. Before the study commenced, all participants provided personal information, completed health questionnaires, disclosed personal medical history, and signed informed consent forms.

**Figure 1.**
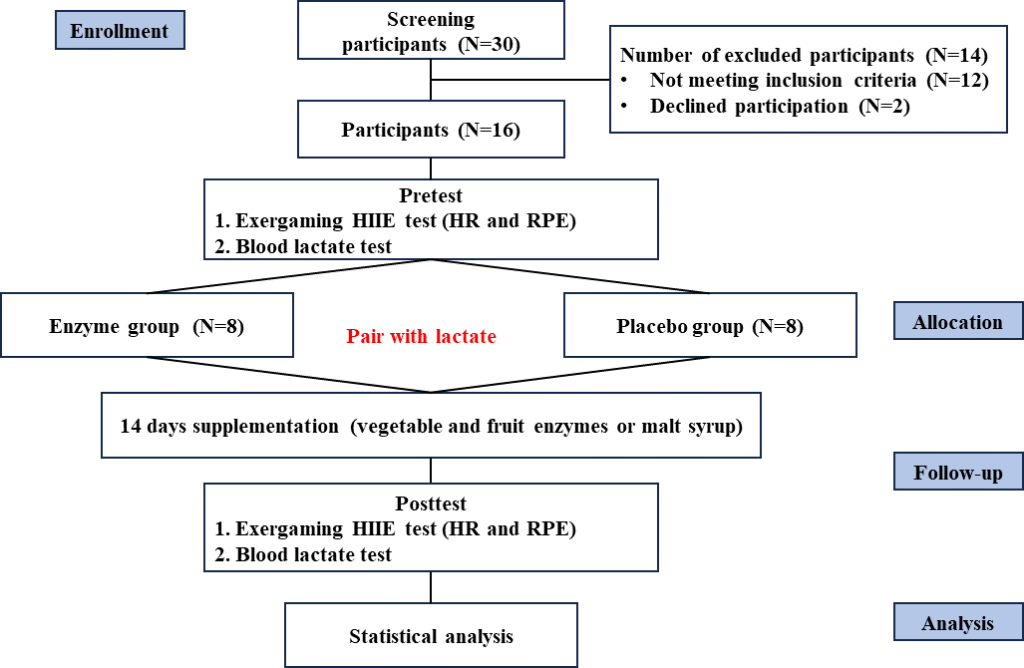
CONSORT (Consolidated Standards of Reporting Trials) and experimental procedure diagram. HIIE: high-intensity interval exercise; HR: heart rate; RPE: rating of perceived exertion.

The 16 participants underwent the exergaming HIIE test as an initial assessment (pretest). Participants engaged in a 5-minute warm-up on a stationary bike, followed by HIIE using Nintendo Switch Ring Fit Adventure. The training method was adapted from previous research [8,9] and consisted of 8 sets of 20 seconds of maximum effort exercise with 30 seconds of complete rest between each set, resulting in a total exercise time of 370 seconds. The HIIE design incorporated training modes targeting the deltoid, pectoralis major, latissimus dorsi, and quadricep muscles in Nintendo Switch Ring Fit Adventure. Blood lactate levels, heart rate (HR), and ratings of perceived exertion (RPE) were recorded before, during, and after exercise, and training load was quantified using training impulse (TRIMP). Participants were matched and divided into 2 groups, the enzyme group and the placebo group, based on their blood lactate levels during HIIE. Each group comprised 8 individuals. Supplementation with vegetable and fruit enzymes or maltodextrin commenced 3 days after the pretest and lasted for a total of 14 days. On the 14th day, following the completion of supplementation, the participants underwent the exergaming HIIE test as a posttest (Figure 1). This study was not preregistered as it was considered a feasibility study.

### Ethical Considerations

The human research ethics committee of the local university approved this study, which was also approved by the human research ethics committee of the National Cheng Kung University, Taiwan (approval NCKU HREC-E-112-419-2). Users volunteered for this study and agreed to participate by signing an informed consent form. To protect the personal data of participants, all participant information has been anonymized and assigned identification numbers. Participation was voluntary following recruitment, and participants were given a small gift at the conclusion as a token of appreciation.

### Supplementation Protocol

After the pretest, the enzyme group consumed 30 mL of vegetable and fruit enzymes (the contents included needle-leaf cherries, cherries, apples, cranberries, blackberries, black currants, blueberries, beets, broccoli, cabbage, carrots, Concord grapes, cranberries, elderberries, kale, oranges, peaches, papayas, parsley, pineapples, raspberries, red currants, spinach, and tomatoes, etc; Enzyme Village) mixed with 150 mL of water twice a day (at breakfast and dinner) for 14 consecutive days. The placebo group followed the same protocol but consumed malt syrup (Amazon) instead until the end of the study. Participants returned to the laboratory each morning to receive the daily supplement, which was administered on site. Following supplementation, participants reported their dinner intake to the researchers, ensuring compliance with the prescribed supplementation regimen.

### Exergaming HIIE Test: Combination of Exergaming and HIIE

**Figure 2.**
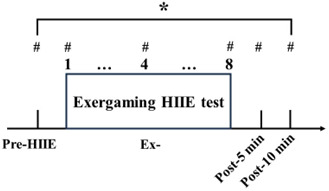
Experimental flowchart. * indicates lactate test. # indicates tests for heart rate and rating of perceived exertion. Ex-: bouts of HIIE; HIIE: high-intensity interval exercise; post–5 min: after 5 minutes of HIIE; post–10 min: after 10 minutes of HIIE.

Participants in this experiment engaged in HIIE using the Nintendo Switch Ring-Con within a laboratory environment. All participants completed pre- and posttest assessments on the same day. The exergame used in this study was Nintendo Switch Fitness Adventure, which ingeniously blends exercise with an adventure narrative to deliver both physical workouts and gaming enjoyment concurrently. This game is noted for its intuitive, user-friendly interface that accommodates players of all ages. It incorporates a specialized fitness ring—a smart accessory that connects to the Nintendo Switch console. The sensor system used 2 Nintendo controllers: 1 mounted on the exercise ring and the other secured to the participant’s thigh to enhance gameplay interaction. Through the Ring-Con, participants engaged in diverse physical activities such as weightlifting, yoga, and aerobic exercises. The fitness ring sensor accurately captures and integrates players’ movements into the game. The gameplay involves unlocking levels and engaging in fitness challenges that are achieved through actual physical activities. It offers a wide range of exercise routines targeting various muscle groups and provides engaging gaming challenges. The exercise protocol included 8 sets of 20-second, high-effort exercises, interspersed with 30-second rest intervals, totaling 370 seconds of active exercise time. Specifically, the fitness game mode used was the Adventure Mode in Ring Fit Adventure, comprising exercises targeting the pectoralis major, latissimus dorsi, deltoid, and quadricep muscles (Figures2  3).

**Figure 3.**
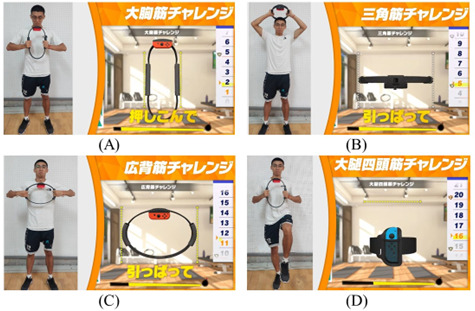
Exercise training model: (A) pectoralis major, (B) deltoid, (C) latissimus dorsi, and (D) quadricep muscles. The images represent the 4 exercises used in this study. (A) shows pressing the fitness ring inward; (B) and (C) depict pulling the fitness ring outward; and (D) illustrates mounting the sensor device on the thigh, which should be raised to approximately 90°.

### Blood Lactate Test

Blood lactate was measured at 5 time points: before exercise, after the fourth and eighth bouts of exercise, and at 5 and 10 minutes after exercise. Blood lactate was analyzed using a Biosen Cline blood analysis system (EKF-diagnostic). Capillary blood samples of 10 μL were collected, added to red blood cell lysis reagent, and stored at low temperature until analyzed. Prior to analysis, instrument standardization and test calibration were performed, and the coefficient of variation was determined to be ≤1.5%. The detection range for blood lactate was 0.5-40 mM [31].

### Exercise Load (TRIMP)

#### Overview

In this study, the exercise load was represented by the TRIMP [32], which was calculated as the product of exercise intensity and duration. To accommodate the convenience of the experiment, 2 different TRIMP calculation methods were used, including % maximum HR (HRmax; objective) and RPE (subjective). At the end of each exercise bout (8 bouts in total) and during the recovery period before the next bout (7 bouts in total), participants were asked to report their RPE, and their HR was recorded. This process was repeated 8 times.

#### % HRmax Calculation Method

During the entire HIIE, the participant’s HR was recorded every 5 seconds using a HR monitor (iHeart Polar) to calculate % HRmax. The block TRIMP method developed by Edwards [33] was used, which divides the exercise intensity into 5 blocks with corresponding weighting factors (Table 1). The weighted score of each block was multiplied by the exercise time (min) and then summed to obtain the exercise load (arbitrary unit [AU]). The calculation formula was as follows: *Exercise load = (Z1 exercise time × 1) + (Z2 exercise time × 2) + (Z3 exercise time × 3) + (Z4 exercise time × 4) + (Z5 exercise time × 5)*.

**Table 1.** The Edwards [33] block training intensity calculation method.

Zone	Intensity (% HRmax^a^), range	Weighted score
Z1	50-60	1
Z2	60-70	2
Z3	70-80	3
Z4	80-90	4
Z5	90-100	5

a HRmax: maximum heart rate.

#### RPE Calculation Method

The TRIMP calculation method of Foster et al [34,35] was used to calculate the exercise load, by multiplying the RPE value of each exercise segment by the exercise time and summing them up. The RPE scale used in this method was the CR-10 version modified by Foster et al [35] based on Borg et al [36] (Table 2). The calculation formula was as follows: *Exercise load (AU) = Borg CR-10 RPE score × exercise time (min).*

**Table 2.** Borg CR-10 rating of perceived exertion (RPE).

Borg CR-10 RPE score	Level of exertion
0	Rest
1	Very, very easy
2	Easy
3	Moderate
4	Somewhat hard
5	Hard
6	Hard
7	Very hard
8	Very hard
9	Very hard
10	Maximal

### Statistical Analysis

All the data were analyzed by SPSS for Windows 20.0 (IBM Corp). Data are expressed as mean (SD) and 95% CI. A mixed design 2-way ANOVA (group×time) was used to compare the variables of lactate response, HR, and TRIMP between 2 groups before and after the 14 days of supplementation. Graphs were generated using GraphPad Prism 8.0 (GraphPad Software). Cohen conventions for ES (Cohen *d*) were calculated by the G*Power 3.1 software program (Heinrich-Heine-Universität), where the ESs of 0.2, 0.5, and 0.8 are considered small, medium, and large, respectively. Statistical significance was set as *P*<.05.

## Results

### Overview

Table 3 outlines the baseline characteristics of participants in the study, divided into the enzyme and placebo groups. The average age of participants was slightly higher in the placebo group (66.50, SD 1.31 y) than the enzyme group (65.75, SD 0.88 y). Heights were similar across both groups, with the enzyme group averaging 160.50 (SD 2.67) cm and the placebo group averaging 160.13 (SD 2.75) cm. The enzyme group members were slightly heavier (mean 56.75, SD 4.27 kg) than those in the placebo group (mean 53.5, SD 3.42 kg), which was also reflected in a higher average BMI (22.02, SD 1.41 kg/m² in the enzyme group vs 20.89, SD 1.71 kg/m² in the placebo group). Regarding exercise habits, both groups engaged in regular physical activity, with the enzyme group exercising on average 3.75 (SD 0.71) days per week and the placebo group exercising slightly more at 4.00 (SD 0.76) days per week. The daily exercise duration was comparable between groups, with the enzyme group averaging 76.25 (SD 41.04) minutes and the placebo group averaging 78.75 (SD 31.82) minutes.

**Table 3.** Participants’ baseline characteristics.

Characteristics	Enzyme group, mean (SD)	Placebo group, mean (SD)
Age (y)	65.75 (0.88)	66.50 (1.31)
Height (cm)	160.50 (2.67)	160.13 (2.75)
Weight (kg)	56.75 (4.27)	53.5 (3.42)
BMI (kg/m^2^)	22.02 (1.41)	20.89 (1.71)
Frequency of regular exercise habits (d/wk within the past year)	3.75 (0.71)	4.00 (0.76)
Daily exercise duration (min)	76.25 (41.04)	78.75 (31.82)

### Enzyme Supplementation’s Impact on Lactate Response in Exergaming Combined With HIIE

**Figure 4.**
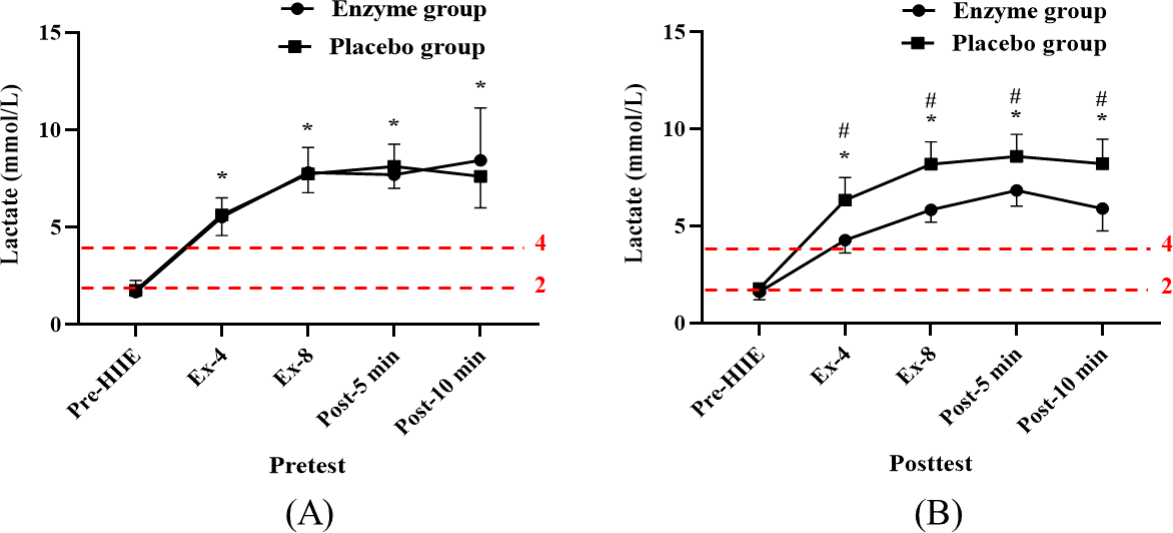
Blood lactate response (A) before and (B) after 14 days of enzyme or placebo supplementation. Data are presented as mean (SD). * indicates a significant difference (*P*<.05) from the pre-exercise value within the group. # indicates a significant difference (*P*<.05) between the groups. Ex-4: fourth bout of HIIE; Ex-8: eighth bout of HIIE; HIIE: high-intensity interval exercise; post–5 min: after 5 minutes of HIIE; post–10 min: after 10 minutes of HIIE.

The results demonstrated that blood lactate levels surpassed 4 mmol/L after the fourth exercise bout, indicating the presence of high-intensity exercise. Additionally, the study examined the effects of 14 days of enzyme or placebo supplementation on blood lactate levels (*F*_1,14_=6.99; *P*=.001). The enzyme group exhibited significantly lower blood lactate levels than the placebo group after the fourth (mean 4.29, SD 0.67; 95% CI 3.56-5.01 vs mean 6.34, SD 1.17; 95% CI 5.61-7.06 mmol/L; ES=−2.14; *P*=.001) and eighth (mean 5.84, SD 0.63; 95% CI 5.14-6.54 vs mean 8.20, SD 1.15; 95% CI 7.50-8.90 mmol/L; ES=−2.56; *P*=.001) exercise bouts, as well as at 5 minutes (mean 6.85, SD 0.82; 95% CI 6.10-7.60 vs mean 8.60, SD 1.13; 95% CI 7.85-9.35 mmol/L; ES=−1.78; *P*=.003) and 10 minutes (mean 5.91, SD 1.16; 95% CI 4.99-6.84 vs mean 8.21, SD 1.27; 95% CI 7.29-9.14 mmol/L; ES=−1.89; *P*=.002) after exercise (Figure 4). These findings suggest that the combination of HIIE and exergaming can lead to high-intensity exercise, and enzyme supplementation can contribute to a reduction in lactate levels.

### Enzyme Supplementation’s Impact on HR in Exergaming Combined With HIIE

**Figure 5.**
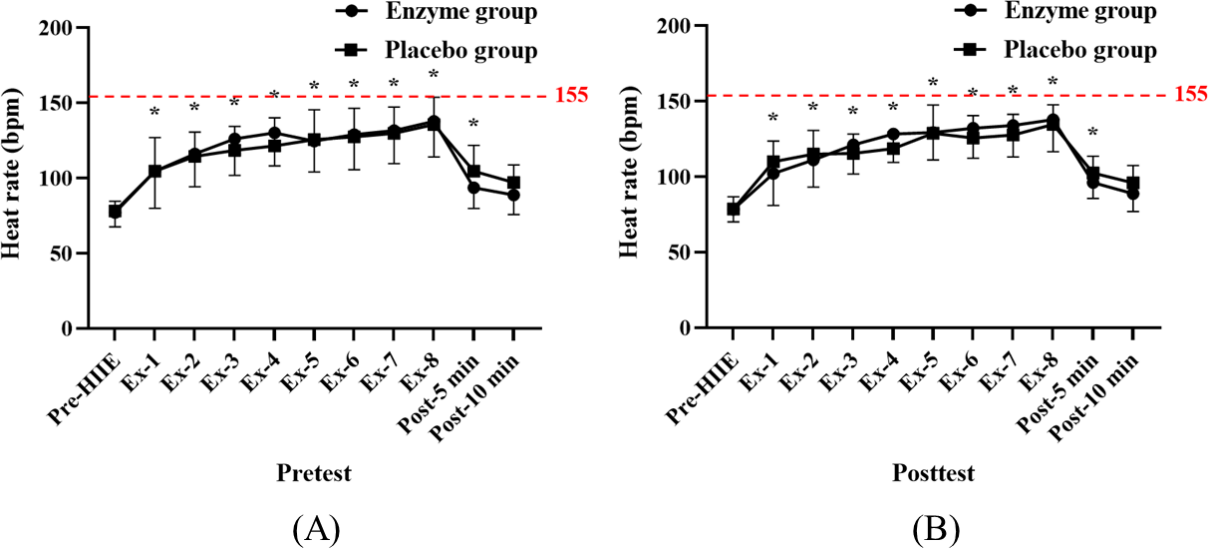
Heat rate response (A) before and (B) after 14 days of enzyme or placebo supplementation. Data are presented as mean (SD). * indicates a significant difference (*P*<.05) from the pre-exercise value within the group. Ex-: bouts of HIIE; HIIE: high-intensity interval exercise; post–5 min: after 5 minutes of HIIE; post–10 min: after 10 minutes of HIIE.

The results demonstrated that during exergaming combined with HIIE, older adult participants experienced a significant increase in HR compared with before exercise (*P*<.05). The estimated HRmax (*220 – age, SD 10*) for older adults was 155 (SD 10) beats per minute (bpm), and the observed HRs during exercise exceeded 85% of the estimated HRmax for both groups. However, there was no significant difference in the average HR of the older adults between the enzyme and placebo groups before and after supplementation. Before supplementation, there was no significant difference in the HRs of the older adult participants between the enzyme and placebo groups during the first (mean 104.63, SD 24.71; 95% CI 86.77-122.48 vs mean 104.63, SD 22.32; 95% CI 86.77-122.48 bpm; ES=0; *P*>.99), second (mean 116.13, SD 21.81; 95% CI 101.54-141.72 vs mean 114.38, SD 16.26; 95% CI 99.79-128.96 bpm; ES=0.09; *P*=.86), third (mean 126.13, SD 24.30; 95% CI 110.53-141.72 vs mean 118.38, SD 15.96; 95% CI 102.78-133.97 bpm; ES=0.38; *P*=.46), fourth (mean 130.13, SD 21.97; 95% CI 114.63-145.62 vs mean 121.38, SD 18.77; 95% CI 105.88-136.87 bpm; ES=0.43; *P*=.41), fifth (mean 124.63, SD 20.49; 95% CI 109.37-139.88 vs mean 125.63, SD 19.73; 95% CI 110.37-140.88 bpm; ES=−0.05; *P*=.92), sixth (mean 128.88, SD 23.34; 95% CI 112.71-145.04 vs mean 127.38, SD 19.06; 95% CI 111.21-143.54 bpm; ES=0.07; *P*=.89), seventh (mean 131.63, SD 22.01; 95% CI 116.56-146.69 vs mean 129.75, SD 17.45; 95% CI 114.69-144.81 bpm; ES=0.09; *P*=.85), and eighth (mean 137.75, SD 23.60; 95% CI 121.78-153.73 vs mean 135.63, SD 18.19; 95% CI 119.65-151.60 bpm; ES=0.10; *P*=.84) sets (Figure 2). Similarly, after supplementation, there was no significant difference in the HRs of the enzyme and placebo groups during the first (mean 102.13, SD 21.21; 95% CI 88.87-115.67 vs mean 109.88, SD 13.64; 95% CI 96.33-123.42 bpm; ES=−0.43; *P*=.40), second (mean 111.13, SD 18.04; 95% CI 98.32-123.93 vs mean 114.88, SD 15.63; 95% CI 102.07-127.68 bpm; ES=−0.22; *P*=.66), third (mean 121.13, SD 19.38; 95% CI 108.63-133.62 vs mean 115.38, SD 12.95; 95% CI 102.88-127.87 bpm; ES=0.35; *P*=.50), fourth (mean 128.25, SD 18.75; 95% CI 116.57-139.93 vs mean 118.5, SD 11.10; 95% CI 106.82-130.18 bpm; ES=0.63; *P*=.23), fifth (mean 129.25, SD 18.12; 95% CI 115.29-143.21 vs mean 128.75, SD 18.68; 95% CI 114.79-142.71 bpm; ES=0.03; *P*=.96), sixth (mean 132.00, SD 19.79; 95% CI 118.70-145.30 vs mean 125.50, SD 14.95; 95% CI 112.20-138.80 bpm; ES=0.37; *P*=.47), seventh (mean 133.88, SD 20.84; 95% CI 120.50-147.25 vs mean 127.63, SD 13.70; 95% CI 114.25-141.00 bpm; ES=0.35; *P*=.49), and eighth (mean 137.75, SD 21.18; 95% CI 124.42-151.08 vs mean 134.63, SD 13.02; 95% CI 121.30-147.95 bpm; ES=0.18; *P*=.73) sets (Figure 5). In summary, the findings indicate that exergaming combined with HIIE leads to a significant increase in HR among older adults. However, there was no significant difference in HR between the enzyme and placebo groups before and after supplementation.

### TRIMP in Enzyme Versus Placebo Groups After Supplementation in Exergaming Combined With HIIE

**Figure 6.**
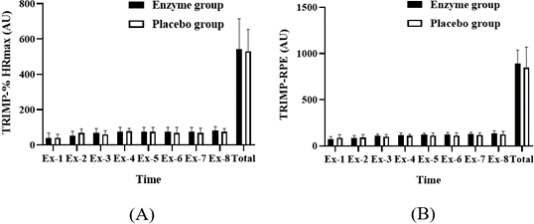
Comparison of (A) objective and (B) subjective training impulse (TRIMP) between the enzyme and placebo groups after supplementation during HIIE with exergaming. Data are presented as mean (SD). AU: arbitrary unit; Ex-: bout of HIIE; HIIE: high-intensity interval exercise; HRmax: maximum heart rate; RPE: rating of perceived exertion.

The TRIMP, representing both objective and subjective training loads, was compared between the enzyme and placebo groups after supplementation. Analysis revealed no significant differences in either the objective (mean 542.5, SD 172.19 vs mean 531.25, SD 123.34 AU; ES=0.08; *P*=.88) or subjective training loads (mean 895, SD 143.73 vs mean 847.50, SD 223.46 AU; ES=0.25; *P*=.62) between the groups (Figure 6). This suggests that the supplementation did not significantly alter the perceived intensity or effort of the HIIE when combined with exergaming.

## Discussion

### Principal Findings

The study investigated the effects of enzyme supplementation on lactate response and HR in older adult individuals engaging in a combination of exergaming and HIIE. The results indicated that enzyme supplementation significantly reduced blood lactate levels after exercise, particularly after the fourth (*P*=.001) and eighth (*P*<.001) exercise bouts, demonstrating the potential of enzymes to mitigate exercise-induced lactate accumulation. Despite a notable increase in HR during the exercise sessions, which surpassed 85% of the estimated HRmax for older adult participants, there was no discernible difference in HR responses between the enzyme and placebo groups, either before or after supplementation. Furthermore, the analysis of TRIMP, encompassing both objective and subjective measures of training load, revealed no significant differences between the enzyme and placebo groups after supplementation. This suggests that although enzyme supplementation may aid in lactate management, it does not significantly impact the overall perceived intensity or cardiovascular demand of HIIE combined with exergaming in older adult individuals.

### Lactate Response in Exergaming

This study contributes valuable insights into the efficacy of fruit and vegetable enzyme supplementation in optimizing exercise outcomes for older adults, particularly when combined with HIIE and exergaming. The notable finding that blood lactate levels surpassed the 4 mmol/L threshold after the fourth exercise bout underlines the high intensity and physiological rigor of the exercise protocol. This study’s emphasis on enzyme supplementation’s impact on blood lactate levels is especially pertinent. Enzyme supplementation significantly lowered blood lactate levels after exercise, as compared to the placebo, after both the fourth (*P*=.001) and eighth (*P*<.001) exercise bouts and at 5 and 10 minutes after exercise (*P*=.003 and *P*=.002, respectively). This observation suggests a potential role of enzyme supplementation in enhancing lactate metabolism, either through its reduction or improved clearance during and after high-intensity exercise. The metabolism-enhancing attributes of fruit and vegetable enzymes, such as bromelain and papain, may facilitate this reduction in lactate accumulation [22,37]. Furthermore, their antioxidant and anti-inflammatory properties could lead to enhanced muscle function, thereby contributing to lower lactate production [38].

Exergaming, when integrated with HIIE, presents an innovative and engaging exercise modality, particularly for older adults. It has been established as an effective and enjoyable exercise option, capable of achieving intensities comparable to traditional exercise forms [16]. This study reinforces the feasibility of exergaming combined with HIIE as a viable strategy for older adults, achieving substantial exercise intensity as evidenced by elevated lactate levels. However, the study is not without limitations. The relatively small sample size and focus on a specific demographic and exercise protocol may restrict the broader applicability of the findings. Further research with larger, more diverse populations is necessary to validate and extend these preliminary results.

### HR Response in Exergaming

Interestingly, although exergaming combined with HIIE effectively elevated physiological parameters such as HR and lactate levels, no significant difference in HR response was observed between the enzyme and placebo groups. This suggests that the subjective perception of effort might not accurately reflect the actual physiological demands of the exercise, echoing previous research [32,33]. In summary, this study illustrates that enzyme supplementation can potentially reduce blood lactate levels during and after high-intensity exercise in an older adult cohort engaged in HIIE combined with exergaming. These findings underscore the value of enzyme supplementation in enhancing metabolic responses and optimizing exercise outcomes. Future research should aim to unravel the underlying mechanisms and investigate the long-term impacts of enzyme supplementation across diverse populations. A deeper understanding of the interplay between nutritional supplementation, exercise modality, and physiological responses is crucial in tailoring effective interventions for optimal exercise performance and overall health promotion.

### TRIMP Response in Exergaming

An additional focal point of our study was the evaluation of TRIMP in relation to enzyme supplementation during HIIE combined with exergaming. TRIMP is a quantifiable measure of training load, incorporating both objective and subjective elements of exercise intensity [32]. In our study, the analysis revealed no significant differences in TRIMP between the enzyme and placebo groups after supplementation. This outcome suggests that enzyme supplementation does not significantly alter the perceived intensity or exertion levels during HIIE with exergaming. This finding aligns with previous studies that have explored the multifaceted nature of TRIMP. For instance, research by Laursen and Jenkins [39] highlighted the complexity of accurately measuring training load, emphasizing the need to consider both physiological and psychological factors. The lack of significant difference in TRIMP in our study could be attributed to the stable physiological responses (HRs and lactate levels) observed across both groups. This observation is consistent with the work of Manzi et al [40], who noted the importance of physiological markers in determining training load, particularly in endurance sports.

Furthermore, the subjective component of TRIMP, which relates to athletes’ perceived exertion, is a crucial aspect of training load assessment [35]. Our study’s findings, where the subjective perception of effort did not significantly differ between the enzyme and placebo groups, resonate with the notion that perceived exertion is a complex interplay of physical and psychological factors [33]. This complexity might explain why enzyme supplementation, primarily impacting physiological responses, did not significantly alter the subjective experience of the training load. The implication of these results is substantial for designing exercise programs for older adults. As suggested by Bethancourt et al [41], understanding and managing training load is crucial in preventing overtraining and optimizing exercise benefits, especially in older adults. The lack of difference in TRIMP between the groups in our study indicates that enzyme supplementation, although beneficial in reducing lactate levels, does not necessarily impact the overall training load as perceived by the participants. This insight is vital for practitioners and researchers in tailoring exercise regimens that are both physiologically effective and psychologically manageable for older adults.

In conclusion, our study contributes to the growing body of knowledge on TRIMP and its applications in exercise science. Although enzyme supplementation shows promise in reducing lactate levels, its impact on the overall training load, as measured by TRIMP, appears to be minimal. Future research should continue to explore this area, considering both physiological and psychological aspects of exercise, to develop comprehensive training strategies for various populations, including older adults.

### Conclusions

This study aimed to evaluate the impact of fruit and vegetable enzyme supplementation on aerobic capacity and blood lactate response in older adults participating in HIIE combined with exergaming. The results demonstrate that enzyme supplementation significantly reduced blood lactate levels after exercise compared to a placebo. This finding is indicative of the potential role of such supplementation in enhancing lactate metabolism during and after high-intensity exercise. Additionally, the integration of HIIE with exergaming has proven to be a novel and effective approach to exercise for older adults, achieving significant physiological intensities while maintaining engagement and enjoyment. However, enzyme supplementation did not exhibit a noticeable effect on HR response or the overall perceived training load, as measured by TRIMP. This suggests that although enzyme supplementation may influence specific physiological responses, such as lactate production and clearance, it does not significantly alter the overall perceived exertion or exercise experience for participants.

These findings contribute to the growing body of literature on the synergistic effects of nutritional supplementation and innovative exercise modalities such as exergaming in the older adult population. They highlight the potential of enzyme supplementation in optimizing exercise outcomes, particularly in reducing lactate accumulation, which is a crucial aspect of high-intensity exercise tolerance. Moreover, the study underscores the feasibility and effectiveness of exergaming combined with HIIE as a strategy to enhance physical activity levels in older adults. The study’s implications extend beyond exercise physiology, offering practical insights for health practitioners, fitness professionals, and researchers in the development of targeted, effective, and enjoyable exercise interventions for older adults. Future research should aim to further elucidate the mechanisms behind enzyme supplementation’s impact on exercise performance and explore the long-term effects of such interventions in a wider demographic.

In summary, this research supports the notion that carefully tailored nutritional and exercise interventions, such as enzyme supplementation combined with HIIE and exergaming, can significantly enhance exercise outcomes in older adults. These interventions hold promise for improving overall health and well-being in this demographic, contributing to the growing field of serious games and their application in health and fitness.

## Supplementary material

10.2196/52231 Checklist 1 CONSORT-EHEALTH checklist (V 1.6.1).

## Abbreviations

AU: arbitrary unit
bpm: beats per minute
ES: effect size
HIIE: high-intensity interval exercise
HR: heart rate
RPE: rating of perceived exertion
TRIMP: training impulse
